# Functionality of Two Origins of Replication in *Vibrio cholerae* Strains With a Single Chromosome

**DOI:** 10.3389/fmicb.2018.02932

**Published:** 2018-11-30

**Authors:** Matthias Bruhn, Daniel Schindler, Franziska S. Kemter, Michael R. Wiley, Kitty Chase, Galina I. Koroleva, Gustavo Palacios, Shanmuga Sozhamannan, Torsten Waldminghaus

**Affiliations:** ^1^LOEWE Centre for Synthetic Microbiology-SYNMIKRO, Philipps-Universität Marburg, Marburg, Germany; ^2^Manchester Institute of Biotechnology, The University of Manchester, Manchester, United Kingdom; ^3^United States Army Medical Research Institute of Infectious Diseases, Frederick, MD, United States; ^4^Defense Biological Product Assurance Office, Frederick, MD, United States; ^5^The Tauri Group, LLC, Alexandria, VA, United States

**Keywords:** DNA replication, secondary chromosome, plasmid, multipartite genome, replication initiation, pathogens, cholera

## Abstract

Chromosomal inheritance in bacteria usually entails bidirectional replication of a single chromosome from a single origin into two copies and subsequent partitioning of one copy each into daughter cells upon cell division. However, the human pathogen *Vibrio cholerae* and other *Vibrionaceae* harbor two chromosomes, a large Chr1 and a small Chr2. Chr1 and Chr2 have different origins, an *oriC-*type origin and a P1 plasmid-type origin, respectively, driving the replication of respective chromosomes. Recently, we described naturally occurring exceptions to the two-chromosome rule of *Vibrionaceae*: i.e., Chr1 and Chr2 fused single chromosome *V. cholerae* strains, NSCV1 and NSCV2, in which both origins of replication are present. Using NSCV1 and NSCV2, here we tested whether two types of origins of replication can function simultaneously on the same chromosome or one or the other origin is silenced. We found that in NSCV1, both origins are active whereas in NSCV2 *ori2* is silenced despite the fact that it is functional in an isolated context. The *ori2* activity appears to be primarily determined by the copy number of the triggering site, *crtS,* which in turn is determined by its location with respect to *ori1* and *ori2* on the fused chromosome.

## Introduction

The generally accepted paradigm of chromosome replication in bacteria is elucidated in *Escherichia coli*. Replication is initiated at a unique singular sequence, the origin of replication (*oriC*) by DnaA, proceeds bidirectionally along the chromosome and ends at the terminus diametrically opposite to *oriC* on the circular chromosome. In *E. coli* and related bacteria, immediate re-initiation of chromosome replication is hindered due to the hemi-methylated status of the sister chromosomes and sequestration of *oriC* by SeqA which has a high binding affinity to hemimethylated *ori* sequences (Lu et al., 1994; Slater et al., 1995; Waldminghaus and Skarstad, 2009). Most bacteria have single chromosomes and follow this general replication paradigm. However, about 10% of bacterial species have more than one chromosome and exhibit some deviation from this norm (Fournes et al., 2018). Among these, *Vibrio cholerae* with chromosome 1 (Chr1, ∼3 Mbps) and chromosome 2(Chr2, ∼1 Mbps) has served as a model system for studies pertaining to multi-chromosome replication mechanisms, and in recent years, an extensive body of information has been accumulated on various aspects of Chr1 and Chr2 replication (Egan et al., 2005; Jha et al., 2012; Val et al., 2014b; Espinosa et al., 2017; Ramachandran et al., 2017).

Chr1 in *V. cholerae* is similar to the *E. coli* chromosome in that the replication follows the same pattern: replication origin, *ori1*, contains multiple DnaA boxes, which are bound by DnaA that unwinds the DNA and initiate replication (Duigou et al., 2006). The similarity is so striking that *V. cholerae ori1* can functionally substitute the *E. coli* replication origin *oriC* (Egan and Waldor, 2003; Demarre and Chattoraj, 2010; Koch et al., 2010; Kamp et al., 2013).

In contrast, the *V. cholerae* Chr2 appears to have an origin that resembles those of low copy number plasmids such as P1 and F (Fournes et al., 2018). The *ori2* contains an array of repeats (iterons) where the Chr2 specific initiator protein, RctB, binds and unwinds the DNA for *ori2* firing (Egan and Waldor, 2003; Duigou et al., 2008) but also exerts a form of negative regulation, termed ‘handcuffing,’ originally discovered in plasmids (Venkova-Canova and Chattoraj, 2011). Although *ori2* has plasmid-like features, Chr2 resembles typical chromosomes in some respects: (1) Participation of SeqA and Dam in regulation of *ori2* (Saint-Dic et al., 2008; Demarre and Chattoraj, 2010; Koch et al., 2010; Stokke et al., 2011). (2) Indispensability of Chr2, unlike plasmids, for cell survival because it harbors essential genes (Heidelberg et al., 2000; Kamp et al., 2013). (3) High level of coordination of replication between Chr1 and Chr2 in order to prevent over replication of Chr2 and ensure a guaranteed inheritance of a single copy of both chromosomes (Baek and Chattoraj, 2014; Val et al., 2016; Ramachandran et al., 2018). This raises the question on how coordination between Chr1 and Chr2 with respect to their timing of replication initiation is achieved given the disparity in their sizes and mechanisms of replication.

Chr1 replication is initiated at the onset of the replication period while initiation of Chr2 is delayed and occurs only when 2/3rd of Chr1 replication has been completed. Since Chr2 is 1/3rd the size of Chr1, both chromosomes consequently terminate their replication roughly at the same time (Rasmussen et al., 2007; Stokke et al., 2011). This termination synchrony appears not to be accidental but is selected for during evolution and is conserved within *Vibrionaceae* despite differing ratios of chromosome sizes (Kemter et al., 2018). This synchrony occurs through the *crtS* (*C*hr2 *r*eplication *t*riggering *S*ite) present on Chr1 that positively regulates *ori2* initiation (Val et al., 2016). Translocation of the *crtS* locus on Chr1, either closer to *ori1* or farther away, resulted in a corresponding shift in Chr2 initiation time as revealed by marker frequency analysis (Val et al., 2016), indicating that the native position of *crtS* sets the timing of Chr2 replication initiation such that its replication terminates synchronously with Chr1 (Kemter et al., 2018). The exact mechanism of *crtS* action remains to be elucidated but may include physical contacts between *crtS* and *ori2* as well as sequestration of Chr2 replication initiator protein, RctB (Baek and Chattoraj, 2014; Val et al., 2016). Recently, the global transcription factor Lrp was shown to bind to the *crtS* site and to facilitate RctB binding (Ciaccia et al., 2018). Heterologous *E. coli* systems have been established based on *ori2* mini-chromosomes demonstrating that *crtS* provided *in trans* increases mini chromosome copy number indicating a positive role played by *crtS* in *ori2* firing (Baek and Chattoraj, 2014; Schallopp et al., 2017; de Lemos Martins et al., 2018). Recently, it was demonstrated that the copy number of *crtS* rather than the act of replicating the *crtS* is critical in the triggering of *ori2* firing (de Lemos Martins et al., 2018; Ramachandran et al., 2018).

In order to assess the differential genetic requirements of Chr1 and Chr2 replication, an artificial single chromosome *V. cholerae* strain has been created by genetic engineering in which *ori1* drives the replication of the fused chromosome (Val et al., 2012). In this strain, designated MCH1, the sequences to the left and right of *ori2* were fused to the terminus of Chr1. In this arrangement the direction in which chromosome arms are replicated is conserved to minimize conflicts between DNA replication and transcription. The MCH1 strain was instrumental in establishing the essentiality of Dam methyltransferase in *V. cholerae* because of its role in *ori2* function which was first shown by Demarre and Chattoraj (2010). This conclusion was further supported by the finding that depletion of Dam leads to spontaneous chromosomal fusion (Val et al., 2014a). In this case, the entire genome is replicated from *ori1* which can tolerate the absence of Dam (Val et al., 2014a).

**FIGURE 1:**
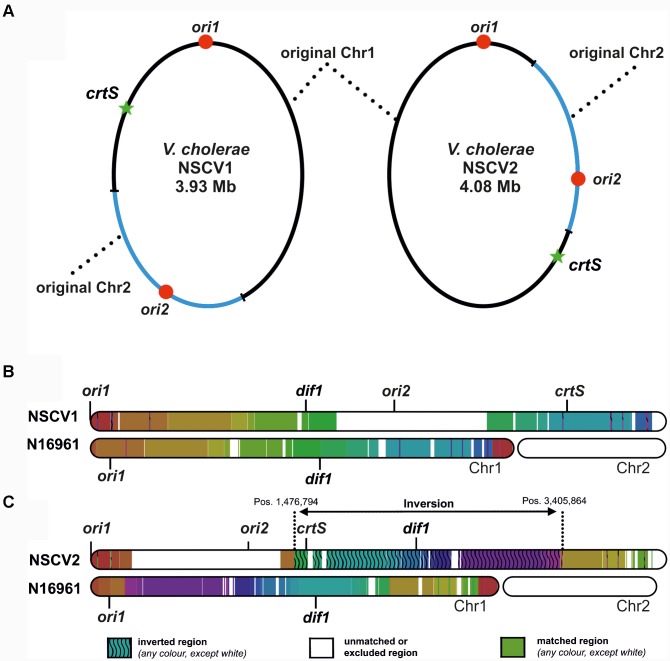
Genetic maps of *V. cholerae* NSCV1 and NSCV2 chromosomes depicting fusion junctions and other genomic features. **(A)** The fused chromosomes of strain NSCV1 (left) and NSCV2 (right) are shown with the original Chr1 and Chr2 in black and blue parts of the ring, respectively. The origins are indicated by red dots and the *crtS* sites by green stars. **(B,C)** Inversion analysis of *V. cholerae* NSCV1 and NSCV2 chromosomes using Smash tool (Pratas et al., 2015). The genome sequences were analyzed for sequence homology with the two-chromosome genome of strain N16961 as reference (accession numbers NC002505 and NC002506). Regions with identical information content are marked by the same color. Inverted regions are indicated by waved lines. White areas represent unmatched regions or Chr2.

Recently, we described two naturally occurring *V. cholerae* strains (NSCV1 and NSCV2) containing both *ori1* and *ori2* on the same chromosome (Chapman et al., 2015; Xie et al., 2017). In these strains, Chr1 and Chr2 are fused at two different locations (Figure 1). The locations of relevant features such as *ori1*, *ori2*, and *crtS* sites are indicated in Figure 1A. In NSCV1, *crtS* is located about 670 kbs away from *ori1* (Figure 1). It is similar to the standard two-chromosome reference strain N16961 with respect to distance, where *crtS* is located 695 kbs away from *ori1*. In NSCV2, the distance between *ori1* and *crtS* is 1,566 kbs due to a large inversion that has occurred around the terminus region of the chromosome (Figure 1C).

This genomic architecture raised interesting questions about whether both origins are functional in the same cell or one or the other *ori* is silenced since in principle a single origin should suffice to replicate the fused chromosome. The strains also allowed us to ask if the chromosomal fusions are maintained without resorting to genome splitting which is the predominant genome configuration in *Vibrionaceae*. We found that in NSCV1 both origins are active in the same cell whereas in NSCV2 *ori2* appears to be silent. Further, these chromosomes appear to be in a locked configuration since even after prolonged continuous growth they remain fused without splitting into two.

## Results

### Activity of *ori1* and *ori2* in *V. cholerae* NSCV Strains

**FIGURE 2:**
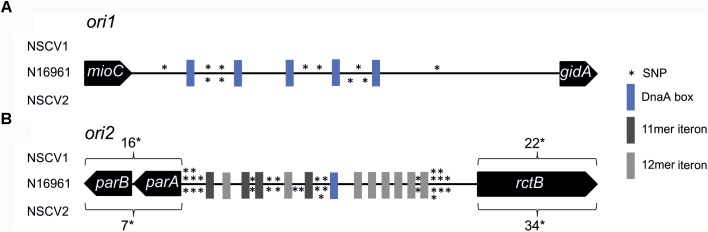
Single nucleotide polymorphism (SNP) analysis of replication origins of *V. cholerae* strains NSCV1 and NSCV2 in comparison to N16961. Genes are indicated as black arrows, RctB binding iterons in dark gray (11 mer iteron) and light gray (12 mer iteron), DnaA boxes in blue and SNPs as stars. The figure is not drawn to scale. Reference sequence: N16961 (Accession # NC002505 and NC002506). Apart from the highlighted SNPs, the sequences are identical. **(A)** Alignment of *ori1*. **(B)** Alignment of *ori2*.

In general, the replication in bacteria relies on one origin of replication for one replicon. Fusion of two replicons, such as seen in NSCV1 and NSCV2, would initially give rise to a chromosome with two functional replication origins where one could be superfluous. This raises the question of whether both, *ori1* and *ori2* are active in the fused chromosomes of NSCV1 and NSCV2, or only one of the origins is active and the other is silent. Inspection of the sequence of the origins and the replication initiator genes revealed no obvious mutational changes that could indicate non-functionality of one or the other of the origins in the two NSCV strains (Figure 2) (Xie et al., 2017). Compared to strain N16961, both strains possess complete *ori1* sequences, with seven SNPs (NSCV1) and four SNPs (NSCV2) spanning the 474 bps long *gidA*-*mioC* intergenic region and none of the DnaA boxes were affected by mutations. The 5,656 bps long *ori2* regions (including genes *parB*, *parA,* and *rctB*) are also intact, with 54 SNPs in NSCV1 and 57 SNPs in NSCV2. Notably, the RctB-binding iteron sequences are not affected by mutations.

**FIGURE 3:**
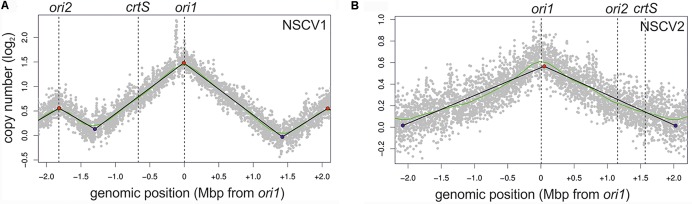
Marker frequency analysis (MFA) of *V. cholerae* NSCV1 **(A)** and NSCV2 **(B)** to assess origin activities. Profiles of genome-wide copy numbers based on Illumina sequencing and read mapping. Gray dots represent log numbers of normalized reads as mean values for 1 kbp windows relative to the stationary phase sample. The genome position is shown as the distance from *ori1*. Vertical dotted black lines mark the locations of replication origins and the *crtS* site. The solid black lines represent the fitting of regression lines and the green line corresponds to the Loess regression (*F* = 0.05). Maxima are highlighted by red and minima as blue dots. Plots of biological replicates are shown in Supplementary Figure S1.

To test the activity of replication origins experimentally, we carried out marker frequency analyses (MFA). It is known that actively growing cells have a higher copy number of *ori* proximal sequences compared to *ori* distal/*ter* proximal sequences. We employed next generation sequencing technology to obtain whole genome sequences of DNAs isolated from logarithmic and stationary phase cultures of NSCV1 and NSCV2 and analyzed the sequence data for marker frequency. Read data from stationary phase DNAs were used for normalization and read mapping plots were created with *ori1* repositioned at the center of the plot to represent bidirectional replication as well as for easy visualization of *ori* activity (Figure 3). Both NSCV1 and NSCV2 exhibited a maximum copy number of reads close to *ori1* and a decreasing gradient on either side of *ori1* moving toward the terminus creating a tent-shape (Skovgaard et al., 2011), indicating an active *ori1* and bidirectional replication in both strains. In strain NSCV1, a local higher marker frequency was observed at the *ori2* position, indicating that *ori2* also is active in this strain. In contrast, no such local peak in marker frequency was found at the *ori2* position in NSCV2 consistent with a silent *ori2*. In addition, an almost 3X lower *ori1/ter* ratio was observed in NSCV2 compared to NSCV1 indicating less or no overlap of replication cycles. The rationale for this interpretation is as follows: On a replicon with overlapping replication, the *ori/ter* ratio would be four since replication is initiated twice before termination can occur. A replicon that initiates only once would consequently have an *ori/ter* ratio of two. Considering that the culture represents a mixed population of cells before and after termination an *ori/ter* ratio of higher than two, as in the case of NSCV1, indicates overlapping replication cycles while values less than two indicate no overlap of replication cycles.

### NSCV1 and NSCV2 Carry a Functional *ori2*

The apparent inactivity of *ori2* in strain NSCV2 raises the question of whether this origin of replication is functional but silenced or non-functional. To answer this question, we cloned the origins into a mini replicon and assessed independent replication in a plasmid backbone. Plasmid pMA135 carries *oriR6K* and can replicate conditionally in *E. coli* strains that provide *in trans*, the replication initiator protein, Pir, from a lambda prophage. In addition pMA135 can be transferred by conjugation from a donor to a recipient (Messerschmidt et al., 2015). In the absence of *λpir*, no exconjugants are obtained unless replication is driven by another fully functional origin of replication. The *ori2* fragments of NSCV1 and NSCV2, including the core *ori2* plus the genes *rctB* and *parAB2*, were cloned into pMA135 independently and the number of exconjugants was enumerated in an *E. coli* strain that does not contain *λpir*. The *ori2* minichromosome constructs from all three strains (N16961, NSCV1, and NSCV2) yielded exconjugants at a frequency of about 1% (10^-2^) of the recipients, as did the positive control F plasmid *ori* (Table 1). Minichromosomes based on *oriII* of strain N16961 have been shown not to integrate into the *E. coli* chromosome (Messerschmidt et al., 2016). To test if the replicons based on NSCV *oriII* are also replicating autonomously, we performed a plasmid isolation procedure for five individual clones for each of the two tested NSCV replicons. In all cases, we were able to isolate the corresponding minichromosomes as evidenced by agarose gel electrophoresis (data not shown), verifying autonomous replication without integration into the primary chromosome. The *oriR6K* replicon by itself did not yield any exconjugant. We conclude that both NSCV1 and NSCV2 carry a functional *ori2.*

**Table 1:** Conjugation efficiencies of replicons with NSCV *ori2* replication origins.

Replicon^†^	Origin of replication	Conjugation^‡^ efficiency
pMA568	N16961*ori2*	5.5 × 10^-3^
pMA739	NSCV1 *ori2*	1.7 × 10^-2^
pMA755	NSCV2 *ori2*	4.3 × 10^-2^
pMA899	F plasmid origin	3.2 × 10^-2^
pMA135	*oriR6K* only	0


*^†^All replicons possess an *oriR6K* and conjugated from *E. coli strain* WM3064 to MG1655. ^‡^Efficiency of one representative experiment is given as ratio of conjugant CFU over total recipient CFU.*

### Genetic Stability of Chromosome Fusions in NSCV1 and NSCV2

**FIGURE 4:**
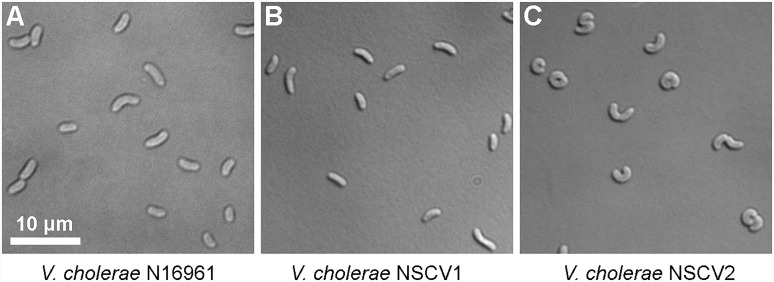
Differential interference contrast (DIC) microscopic images of cells of various *V. cholerae* strains growing in exponential phase. **(A–C)** N16961 represents the typical 2 chromosome *V. cholerae* and NSCV1 and NSCV2 are Chr1 and Chr2 fusion strains.

An active *ori2* in strain NSCV1 could potentially allow the chromosome fusion to be reversed. Similarly, the silent *ori2* in NSCV2 might be inactive only in the context of a fused chromosome; in either case, splitting of Chr1 and Chr2 is conceivable. It was observed that *V. cholerae* with an artificially fused chromosome grew slower compared to the two-chromosome parental strain indicating a negative fitness burden on the bacterium (Val et al., 2012). Similarly, we observed an increased doubling time of strains NSCV1 (20 ± 0.5 min) and NSCV2 (29 ± 1.3 min) compared to the two-chromosome strain N16961 (16 ± 0.2 min) (Supplementary Table S4). In addition, microscopic examination showed that strain NSCV2 cells exhibited a distinct phenotype. While the two-chromosome reference strain N16961 and NSCV1 cells are comma-shaped as typical for *V. cholerae*, NSCV2 cells are much more curled and occasionally S-shaped (Figure 4). It is unknown whether this phenotype is related to the chromosome fusion.

It is conceivable that the slower growth of the fused chromosome strains may have negative fitness value leading to instability, thus promoting genome splitting. On the other hand, if the fused chromosome were under a positive selection pressure to remain fused even at a greater cost in terms of slower growth rate or if they are locked in the fusion configuration due to genetic defects that prevent splitting, then the fusion would be stable even after long term continuous culturing. This led us to test whether the two NSCV strains potentially could revert back to a two-chromosome arrangement upon prolonged continuous culturing. If splitting of the fused chromosomes occurs and if this splitting leads to a fitness advantage due to increased growth rates one would expect two-chromosome clones to appear in a population of NSCV1 and NSCV2 and these clones could potentially replace the fused chromosome cells after prolonged growth by clonal expansion. To test this hypothesis, we cultured the two NSCV strains for 16 days in 100 ml of liquid medium with replenishment of fresh medium every 24 h resulting in approximately 160 generations of growth in total. To examine if the NSCV strains reverted back to a two-chromosome arrangement we isolated DNA from long-term grown cells and performed long-read DNA sequencing using the PacBio technology to be able to detect long range chromosomal rearrangements such as the chromosomal fusion junctions/or junctions (∼24–51 kbs) of the split chromosomes. A *de novo* genome assembly led to one single contig for both NSCV1 and NSCV2 reflecting the original one-chromosome configuration. In conclusion, the chromosome fusions in NSCV1 and NSCV2 appear to be stable and chromosome splitting is not a frequent event or the fused state is probably under positive selection pressure.

### Replication Origins Are Active in an Engineered System Resembling the NSCV Arrangement

Why is *ori2* of strain NSCV2 silenced while it is active in NSCV1? One obvious difference between the two strains is the differential positioning of the two replication origins *ori1* and *ori2* to one another. While the distance between *ori1* and *ori2* is 1.828 Mbps in NSCV1 it is only 1.118 Mbps in the genome of NSCV2.

**FIGURE 5:**
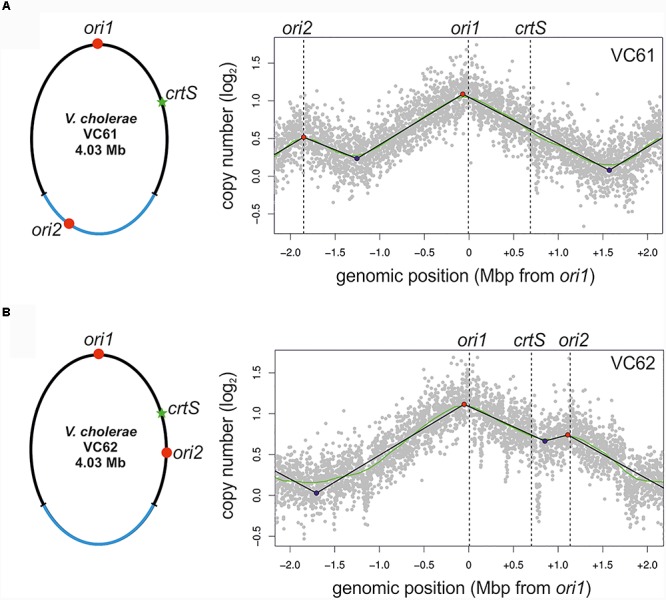
Marker frequency analysis of engineered single chromosome *V. cholerae* strains VC61 **(A)** and VC62 **(B)**. Left panel: Genomic maps of VC61 (analogous to NSCV1) and VC62 (analogous to NSCV2) showing the respective locations of *ori1*, *ori2*, and *crtS*. Right panel: Profiles of genome-wide copy numbers based on Illumina sequencing. Gray dots represent log numbers of normalized reads as mean values for 1 kbp windows relative to the stationary phase sample. Vertical dotted black lines mark the locations of replication origins of replication and the *crtS* sites. The solid black lines represent the fitting of regression lines and the green line corresponds to the Loess regression (*F* = 0.05). Maxima are highlighted by red and minima as blue dots. Plots of biological replicates are shown in Supplementary Figure S1.

To test experimentally, if the *ori* positioning of NSCV1 and NSCV2 influences the initiation outcome, we re-constructed the *ori1* to *ori2* arrangement in a genetically accessible system since NSCV1 and NSCV2 are recalcitrant for genetic manipulations. To accomplish this, we transferred a functional *hapR* to *V. cholerae* strain MCH1 to render it naturally competent (Lo Scrudato and Blokesch, 2012, 2013). Strain MCH1 was derived from the prototype *V. cholerae* strain N16961 by fusion of the two chromosomes with a deletion of *ori2* (Val et al., 2012). We inserted a copy of *ori2* including the flanking genes *parAB* and *rctB* into MCH1 at positions analogous to NSCV1 and NSCV2 with respect to the distance from *ori1* giving rise to strains VC61 and VC62, respectively, and performed marker frequency analysis of exponentially grown cultures (Figure 5).

The MFA plot of strain VC61 resembled the pattern seen in NSCV1 (Figure 5A, compared to Figure 3A). In contrast, a clear local peak of marker frequency was seen at the *ori2* position of strain VC62 (Figure 5B). Thus, even though the origin arrangement is similar in NSCV2 and VC62, the *ori2* copy appears to be active only in the engineered strain VC62 and not in NSCV2. To test if the *ori2* positioning at the NSCV2 position has more negative effect on growth compared to the *ori2* insertion at the NSCV1 position we measured the doubling times of strains VC61 (26 ± 0.1 min) and VC62 (27 ± 0.5 min) (Supplementary Table S4). The difference in doubling time was only marginally affected suggesting that the differences in DNA replication between the two strains do not result in severe impairment in growth. We conclude that an *ori2* insertion at positions analogous to those in the NSCV strains relative to *ori1* can be active as determined by MFA.

### Dam Methyltransferase Is Functional in NSCV Strains

We have shown above that *ori2* in strain NSCV2 is functional but appears to be not active. One potential cause of *ori2* silencing could be an inactive Dam methylation system. Methylation of the adenine within the Dam recognition site ‘GATC’ present at *ori2* locus is a prerequisite for *ori2* activity (Demarre and Chattoraj, 2010). Sequence analyses showed intact *dam* genes in both strains (Xie et al., 2017). The methylation status of GATC sites can be analyzed by the differential sensitivities of the genomic DNA to *Dpn*I (cleaves only methylated/hemimethylated GATC sites), *Dpn*II (cleaves only unmethylated GATC sites) and *Sau*3A1 (cleaves both unmethylated and methylated GATC sites) restriction enzymes.

**FIGURE 6:**
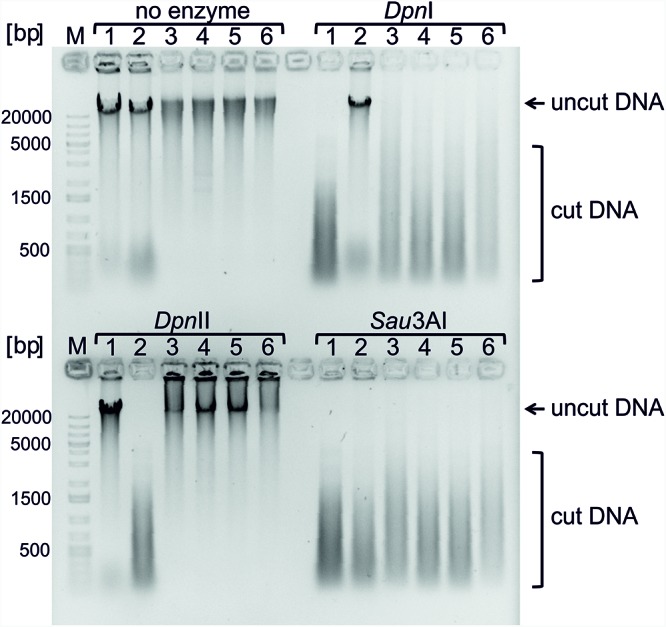
Testing Dam methylation sensitivities of genomic DNAs of strains *V. cholerae* NSCV1 and NSCV2. Restriction digestion of 1 μg of genomic DNA using the indicated enzymes was carried out and the various strains are as follows: 1. *E. coli* MG1655; 2. *E. coli* MG1655Δ*dam*; 3 and 4. *V. cholerae* NSCV1; 5 and 6. *V. cholerae* NSCV2. *Dpn*I cleaves methylated GATC sequences. *Dpn*II cleaves only unmethylated GATC sequences. *Sau*3AI cleaves GATCs independent of the methylation state. DNA cleavage is evident from the disappearance of high molecular weight band and appearance of low molecular weight streak of DNA.

Genomic DNAs of NSCV1 and NSCV2 were sensitive to *Dpn*I and *Sau*3A1 and resistant to *Dpn*II, indicative of full methylation at GATC sites (Figure 6). This result was further confirmed by the PacBio sequence data. In PacBio SMRT (Single Molecule Real Time) sequencing, presence of modified base (A in GATC) in the DNA template results in a delayed incorporation of the corresponding T nucleotide, i.e., longer inter-pulse duration (IPD) compared to template lacking the modification (Flusberg et al., 2010). These kinetic measurements create specific signatures for different types of base modifications. Analyses of the PacBio sequence data for modified bases indicated that both NSCV1 (38571/38572 sites) and NSCV2 (37573/37590) have fully methylated (>99.99%) GATC sites. We conclude that Dam is functional in both NSCV strains and a lack of methylation cannot explain the inactivity of *ori2* in NSCV2.

### *crtS* Sites of NSCV Strains Are Functional

**FIGURE 7:**
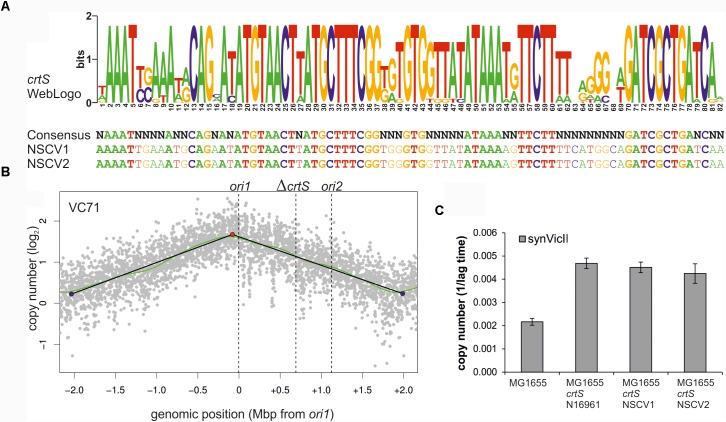
*crtS* sites of NSCV strains are functional. **(A)** An alignment of *crtS* sites from 13 sequenced *Vibrio* genomes was used to calculate a WebLogo (top panel). The height of the nucleotides in each position represents the measure of conservation. All positions with 100% sequence conservation are conserved in the *crtS* sequences of NSCV1 and NSCV2 as well (indicated in bold, lower panel) (Kemter et al., 2018). **(B)** Marker frequency analysis of *V. cholerae* strain VC71 lacking a functional *crtS* site. Gray dots represent log numbers of normalized reads as mean values for 1 kbp windows relative to the stationary phase sample. The genome position is shown as the distance from *ori1*. Vertical dotted black lines mark the locations of replication origins of replication and the *crtS* sites. The solid black lines represent the fitting of regression lines and the green line corresponds to the Loess regression (*F* = 0.05). Maxima are highlighted by red and minima as blue dots. Plots of biological replicates are shown in Supplementary Figure S1. **(C)** *crtS* sites of NSCV1 and NSCV2 increase the copy number of *ori2*-based mini-chromosomes in *E*. *coli*. *E. coli* strains harboring the *ori2*-based mini-chromosome synVicII and chromosomal insertions of *crtS* from different *V. cholerae* strains as indicated were grown in LB medium with 500 μg/ml ampicillin in a 96-well plate at 37°C. As strains with a lower replicon copy number have a longer lag period to initiate growth, the value 1 divided by the time to reach an OD_600_ ≥ 0.1 was used as measure for the replicon copy number. Values are the mean of three biological replicates with indicated standard deviation. Growing the strains in standard concentrations of ampicillin did not show any difference between *wild type* and *crtS* carrying strains as expected (data not shown).

Another possibility for *ori2* silencing in NSCV2 is through alterations of *crtS* activity. This short DNA sequence is found on Chr1 in all available whole genome sequences of two-chromosome strains of *Vibrionaceae* and its replication triggers *ori2* firing on Chr2 (Baek and Chattoraj, 2014; Val et al., 2016; Kemter et al., 2018). Consequently, a non-functional *crtS* could lead to an inactive *ori2*. To decipher the functionality of *crtS* sites in NSCV1 and NSCV2 *in silico*, we extracted the respective sequences and aligned them to the consensus sequence we established recently (Figure 7A) (Kemter et al., 2018). Notably, all highly conserved parts of the *crtS* sequence are also conserved in the *crtS* sequences of NSCV1 and NSCV2 (Figure 7A). To test the functionality of *crtS* in the context of a fused chromosome experimentally, we deleted the *crtS* in strain VC62 (resulting in strain VC71) and performed marker frequency analysis (Figure 7B).

As expected, no local peak of copy number increase was seen at the *ori2* position of the Δ*crtS* strain VC71 in contrast to the MFA of the parental strain VC62 (compare Figures 5B, 7B) confirming the necessity of a functional *crtS* site for *ori2* firing. Incidentally, the doubling time of this strain is not much different (25 ± 0.6 min) from that of VC61 and VC62 (Supplementary Table S4). To analyze the functionality of the *crtS* sites from NSCV1 and NSCV2, we inserted these sequences into the genome of *E. coli* and transformed the corresponding strains with the *ori2*-based minichromosome synVicII. The rationale behind this approach is the observation of a copy number increase of an *ori2*-based minichromosome in *E. coli* strains carrying a functional *crtS* site (Baek and Chattoraj, 2014; Kemter et al., 2018). The copy number of *ori2* minichromosomes was measured in *E. coli* strains either carrying the *crtS* sites of strain N16961, NSCV1, NSCV2 or no *crtS* (Figure 7C). In this case, we tested the resistance level of the respective strains to ampicillin as an indirect measure of the minichromosome copy number as described previously (Schallopp et al., 2017). The *crtS* sites of both NSCV strains increased the copy number of the *ori2* minichromosome compared to the strain lacking any *crtS* site and to a similar extent as the *crtS* site from the two-chromosome *V. cholerae* strain N16961 used as positive control (Figure 7C). We conclude that *crtS* sites in NSCV1 and NSCV2 are functional in a heterologous system and a defective *crtS* might not explain the silent *ori2* in strain NSCV2.

### Relative *ori1*, *ori2*, and *crtS* Locations Determine *ori2* Activity in NSCV Strains

**FIGURE 8:**
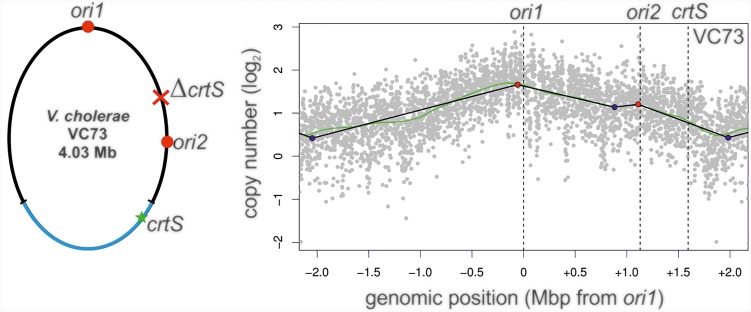
Marker frequency analysis of *V. cholerae* strain VC73. Left panel: Genomic map of VC73 showing the locations of *ori1*, *ori2*, and *crtS*. Right panel: Profile of genome-wide copy numbers. Gray dots represent log numbers of normalized reads as mean values for 1 kbp windows relative to the stationary phase sample. The genome position is shown as the distance from *ori1*. Vertical dotted black lines mark the locations of replication origins of replication and the *crtS* sites. The solid black lines represent the fitting of regression lines and the green line corresponds to the Loess regression (*F* = 0.05). Maxima are highlighted by red and minima as blue dots. Plots of biological replicates are shown in Supplementary Figure S1.

In a canonical two-chromosome *V. cholerae*, replication of the *crtS* on Chr1 triggers the initiation at *ori2* on Chr2. This scenario can in principle be analogous in a fused chromosome such as NSCV1 in which *ori1*, *ori2* and *crtS* are present on the same molecule. Here the *crtS* is located about 677 kbps away from *ori1* and is replicated first with replication forks originating at *ori1* which subsequently triggers *ori2* firing, which is further downstream of *crtS*. The organization is different in strain NSCV2 where the *crtS* lies about 416 kbps downstream of *ori2*. Thus, in NSCV2, *ori2* precedes *crtS*. This arrangement is the consequence of a large inversion of the Chr1 part of the genome (Figure 1). The replicative outcome of this arrangement is difficult to predict considering the known triggering role of *crtS*; i.e., *ori2* being replicated first by replication forks originating at *ori1*, subsequently upon duplication of the *crtS* site. In other words, *crtS* can also retrospectively trigger replication of already replicated *ori2* that *crtS* replication is supposed to trigger in the first place. To test this scenario, we moved the *crtS* site in strain VC62 to the position analogous to strain NSCV2 (resulting in strain VC73). VC73 did not exhibit any significant difference in doubling time compared to other strains (26 ± 0.2 min) (Supplementary Table S4). Respective MFA analysis revealed an active *ori2,* suggesting that an *ori2* copy on a fused chromosome can be triggered by a *crtS* site located downstream relative to *ori2*, contrary to what was observed in NSCV2 (compare Figures 3B– 8). The peak at the *ori2* position is not very strong but it is important to note that the fitting of regression lines is automated based on the maxima found within the mapped reads (Kemter et al., 2018). There is no pre-selection of *ori* positions and the fact that the peak is detected at the *ori2* position by the computational fitting model implies that it is an active origin as also seen in the biological replicate (Supplementary Figure S1F).

## Discussion

*Vibrio cholerae* has partitioned its genome between a true bacterial chromosome and a “domesticated” plasmid replicon (Heidelberg et al., 2000; Venkova-Canova and Chattoraj, 2011; Val et al., 2014b). Unlike most bacteria, this two-replicon arrangement is conserved within the family of *Vibrionaceae* (Okada et al., 2005; Val et al., 2014b; Ramachandran et al., 2017). It was postulated that the bipartite genome in *Vibrio* species enables varying the copy numbers of both chromosomes in a niche-specific manner under certain environmental conditions as an adaptation strategy (Heidelberg et al., 2000; Schoolnik and Yildiz, 2000; Srivastava and Chattoraj, 2007). Alternatively, the two-chromosome setup in *Vibrio* species can be considered as an adaptive feature that enables rapid genome duplication if multiple chromosomes are replicated simultaneously. The latter assumption fits with the observation that *Vibrio* species is one of the fastest growing bacteria known, a feature that has led to a recent proposal for using the non-pathogenic *V. natriegens* as the new workhorse for bioengineering (Weinstock et al., 2016; Dalia et al., 2017; Hoffart et al., 2017). However, it brings up the question of how the two replicons are coordinating their replication and regulate over or under replication in order to ensure inheritance of the just one copy of the full complement of the genome into daughter cells upon cell division. Furthermore, the evolutionary driving force (or forces) that led to the two-chromosome setup in *Vibrionaceae* remain speculative. One way to better understand the evolutionary significance of this unique feature of *Vibrio* species is to study naturally occurring exceptions to the two chromosome rule: strains which have evolved into a single chromosome by chromosomal fusion. We have previously identified two Natural Single Chromosome *Vibrio* (NSCV) *cholerae* strains (Chapman et al., 2015; Johnson et al., 2015; Xie et al., 2017) and in this study we addressed the functionality and activity of the two origins of replication on the same chromosome.

Fortuitously, the two NSCV strains have different fusion junctions and other genetic rearrangements that provided an opportunity to compare and contrast their *ori* functionality. We used marker frequency analysis as an indirect measure of *ori* activity and found *ori1* to be active in both strains. In strain NSCV1, we detected an additional, replication activity peak at the *ori2* locus indicating that this second origin is active as well. Since the MFA experiments are population based, we cannot exclude that some cells use *ori1* only and other cells *ori2* only to replicate the fused chromosome. However, we consider it more likely that both origins are firing within the same cell. Interestingly, the copy number ratio of *ori2/ter* was lower than for *ori1/ter* consistent with a time delayed initiation of *ori2* on the fused chromosome. Such an origin initiation differential has also been observed in conventional two-chromosome *Vibrio* strains resulting in synchronous termination of replication of the two chromosomes despite their different sizes (Rasmussen et al., 2007; Stokke et al., 2011; Kemter et al., 2018). Conservation of the orchestrated replication timing of the two origins in NSCV1 indicates that the single-chromosome setup does not lead to any origin interference. In two-chromosome *Vibrio* species, replication of the *crtS* site located on Chr1 has been shown to trigger *ori2* firing (Baek and Chattoraj, 2014; Val et al., 2016; Ramachandran et al., 2018). In *V. cholerae* N16961, the time between *crtS* replication and *ori2* initiation corresponds to the time the replication fork needs to replicate about 200 kbps of DNA (Val et al., 2016). Considering a similar delay in strain NSCV1, *ori2* would have fired long before replication fork originating at *ori1* reaches it since the distance between *crtS* to *ori2* is more than 1 Mbps. As a consequence, replication forks originating at *ori1* and *ori2* will meet at some position of the fused chromosome, the timing of which will dependent on *ori1* location and its regulation just as it occurs in two-chromosome *V. cholerae*.

The scenario is much different in NSCV2. Here, *ori2* is replicated before *crtS* by replication forks originating at *ori1* (Figure 1A). Intuitively, this would lead to a chaotic replication perturbance because *ori2* firing precedes *crtS* duplication. In the two-chromosome context, *crtS* duplication occurs first which then triggers *ori2* firing. Hence, if replication of the *crtS* triggers *ori2* initiation also in this genomic arrangement it would happen on two copies of the already replicated *ori2*. In addition, the replication forks coming from this newly initiated *ori2* copies have the potential to replicate the *crtS* in just a few minutes because of their close proximity and could potentially lead to additional rounds of *ori2* initiation and thus an uncontrolled *ori2* firing. If this were to happen, not only replication control at *ori2* would be severely perturbed but also *ori1* functioning can be interfered because *ori1* might be replicated passively from replication forks, coming from *ori2*. Interestingly, what we observed in strain NSCV2 is not a replication out of control but instead a simple silencing of *ori2* activity. Our data clearly demonstrate that *ori2* of NSCV2 is functional in an isolated context as shown by its ability to drive replication of a mini-chromosome in *E. coli.* In addition, critical factors involved in regulation of *ori2* appear to be fully functional in NSCV2, namely the Dam methylation system and the *crtS* site. Paradoxically, *ori2* is not used to initiate DNA replication in the fused chromosome strain, NSCV2.

A recent study offers a simple explanation for our observation on *ori2* silencing in NSCV2 (Ramachandran et al., 2018). These authors showed that it is the doubling of the *crtS* dosage rather than the process of replicating the *crtS* that triggers *ori2* initiation which results in an even number of *crtS* and *ori2* copies. This tendency of the regulatory system to produce similar copy numbers of *crtS* and *ori2* have also been found in engineered systems with multiple *crtS* sites (de Lemos Martins et al., 2018). If the *crtS* site is duplicated before *ori2* as it is the case in two-chromosome *V. cholerae* strains, the *ori2* copy number will be lower compared to *crtS* and consequently the initiation at *ori2* will be triggered to restore *crtS*/*ori2* copy number balance. If the *crtS* site is replicated after *ori2* has been copied by replication forks coming from *ori1* as in the case of NSCV2, there is no need to initiate at *ori2* because the copy number of *crtS* and *ori2* are in balance already. The lack of initiation at *ori2* in NSCV2 is therefore fully consistant with and confirmatory to the findings of the aforementioned studies (de Lemos Martins et al., 2018; Ramachandran et al., 2018). However, in contrast to the expectation of the *crtS*-to-*ori2* copy control model, we observed that *ori2* is active in the engineered strain VC73 in which the *crtS* lies downstream of *ori2* analogous to the arrangement in NSCV2 (Figure 8). However, it is important to note that although the positioning of *ori1*, *ori2* and *crtS* are similar in strains NSCV2 and VC73, the genomic contexts of *ori2* and *crtS* are entirely artificial compared to their native positions and furthermore, VC73 does not share the large chromosomal inversion found in NSCV2. More precisely, in VC73, *ori2* lies in a region of the original Chr1 and *crtS* within a region of the original Chr2 (Figure 8, left panel). The discrepancy in *ori2* firing between the naturally occurring NSCV2 and the engineered strain VC73 may therefore be explained by their different genomic context.

One interesting question that arises is if the two *V. cholerae* strains with fused chromosomes are evolutionarily stable. It could also be that the fusion occurred only transiently as an artifact of lab cultivation or during the original strain isolation from a patient sample. In fact, chromosome fusions in *V. cholerae* have been observed previously to occur frequently within a population but the two chromosome configuration seems to provide a selective advantage leading to rapid elimination of the fused chromosomes cells from the population (Val et al., 2014a). Based on this observation, we grew the NSCV strains for about 160 generations and expected a splitting of the fused chromosome that may provide a selective advantage thereby eliminating the cells with single chromosomes from the population. Contrary to the expectation, we found that cells retained the fused chromosomes indicating that they are in fact, locked in this configuration and not easily revertible. An alternative explanation would be that the fused state is under positive selection pressure. It remains to be seen what the significance of the fused chromosome status in NSCV1 and NSCV2 is, in contrast to vast majority of other *V. cholerae* strains where two chromosome status is the norm. In any case, the single-chromosome *V. cholerae* appears to be more frequent than expected as yet another NSCV strain was discovered recently (Yamamoto et al., 2018). We expect future studies of NSCV strains which are a deviation from the norm, would to lead to a better understanding of why most *V. cholerae* carry their genome split into two as the norm.

## Materials and Methods

### Strains, Plasmids, Oligonucleotides, and Growth Conditions

All strains, plasmids and oligonucleotides used in this study are listed in the Supplementary Tables S1–S3. Unless indicated otherwise, the bacterial cells were grown in LB medium at a temperature of 37°C. Antibiotic selection was performed at the following concentrations, if not indicated differently: Ampicillin 100 μg/ml, Kanamycin 35 μg/ml for *E. coli* and 70 μg/ml for *V. cholerae*, Spectinomycin 100 μg/ml, Gentamicin 20 μg/ml, Chloramphenicol 35 μg/ml. Where needed, diaminopimelic acid (DAP) was added to the medium at a concentration of 300 μM. To determine doubling times, cells were grown in LB medium in a 96-well plate at 37°C in a microplate reader (Infinite M200 pro multimode microplate reader, Tecan). OD_600_ was measured every 5 min for 18 h. Doubling times were calculated in exponential phase for OD_600_ values between 0.01 and 0.1. For continues cultivation of NSCV strains to investigate potential chromosome splitting strains were inoculated in the morning and grown in 100 ml liquid medium for 24 h. The next morning, new flasks were inoculated 1:1,000 from the previous cultures. After 4 working days (on day 5), 1 ml samples of the cultures were frozen at -80°C as glycerin stocks. Two days later, new flasks were inoculated from these glycerin stocks and the procedure was continued for a total of 16 days. Before sequencing, these were re-streaked on TCBS medium to verify that it is *V. cholerae* and to obtain single colonies. For each strain one single colony was used to inoculate a culture for DNA isolation and sequencing.

### Construction of Replicons and Strains

NSCV minichromosomes were constructed by PCR amplifying the respective origins of replication using the primers 1227/1228 on *V. cholerae* NSCV1 genomic DNA for pMA739 or *V. cholerae* NSCV2 genomic DNA for pMA755. The vector pMA135 was digested with *Asc*I and the PCR products were integrated in the linearized vector using Gibson Assembly (Gibson et al., 2009) used to transform *E. coli* WM3064. For construction of chromosomal integrations and deletions, integration cassettes were assembled by using the MoClo system as described previously (Weber et al., 2011; Milbredt et al., 2016; Schindler et al., 2016). The *ori2* insertion cassettes on pMA735 and pMA736, as well as the *crtS* deletion and insertion cassettes on pMA748 and pMA749 were assembled by MoClo reactions using the plasmids indicated in Supplementary Table S2, which themselves were assembled by MoClo reactions of the respective PCR products into the respective backbones (primers, templates, and backbones indicated in Supplementary Table S2). The linear cassettes were released by restriction enzyme digestions with *Bsa*I. Triparental mating was performed to deliver the plasmids pUXBF13 and pGP704-mTn7-hapR_ATN from *E. coli* S17-1 *λpir* to *V. cholerae* MCH1 (Bao et al., 1991; Meibom et al., 2005; Val et al., 2012). The created strain *V. cholerae* VC49 was naturally competent and by transforming it with the *ori2* insertion cassettes released from pMA735 or pMA736, respectively, followed by transformation with pBR-*flp* and flippase reaction strains VC61 and VC62 were created (De Souza Silva and Blokesch, 2010). *V. cholerae* strain VC71 was constructed by deletion of the *crtS* sequence by transforming *V. cholerae* VC49 with the *crtS* deletion cassette from pMA748 and a flippase reaction to excise the resistance marker. Subsequently, strain VC71 was transformed with the *crtS* insertion cassette from pMA749 and the flippase reaction was performed to create strain VC73. To construct plasmid pMA449, *crtS* was amplified with primers 1439/1440 from gDNA of *V. cholerae* NSCV1 and for pMA450 with primers 1439/1440 from gDNA of *V. cholerae* NSCV2. PCR products were assembled in pMA349 by MoClo assembly as described (Schindler et al., 2016) and used to transform *E. coli* TOP10 cells. Details of further MoClo assemblies are provided in Supplementary Table S2. Assemblies were used to transform *E. coli* DH5α λpir. The derived integration cassettes were cut out with BsaI, integrated into the chromosome of *E. coli* AB330 and transferred to *E. coli* MG1655 per P1-transduction. FRT recombination was used to remove the resistance marker.

### Sequence Comparison

Sequences from *V. cholerae* N16961, NSCV1 and NSCV2 were compared by multiple sequence alignment using Clustal Omega (Goujon et al., 2010; Sievers et al., 2011) to find single nucleotide polymorphisms. To analyze the whole genomes of the *V. cholerae* strains NSCV1 and NSCV2 for sequence homology, the alignment free chromosome comparison tool SMASH (Pratas et al., 2015) was used with a minimum block size setting of 5,000 bp.

### Preparation of Genomic DNA From Bacteria

The desired amount of culture (between 0.1 ml and 5 ml) was mixed 1:1 with ice-cold killing buffer and centrifuged at maximum speed and 4°C for 3 min. The pellet was resuspended in 300 μl TE, 40 μl SDS and 3 μl 0.5 M EDTA. After 5 min incubation at 65°C, 750 μl Isopropanol were added and the sample was centrifuged 5 min at maximum speed. The pellet was resuspended in 500 μl TE and 2 μl RNAse were added. After 30 min at 37°C, 2 μl of Proteinase K were added and incubated for another 15 min. The sample was purified via twofold Phenol-Chloroform extraction and precipitated over night at -20°C by mixing it with 1 ml pure ethanol and 40 μl 3 M sodium acetate.

The next day, DNA was spinned down at maximum speed for 10 min, the pellet was washed with 70% ethanol and centrifuged another 10 min. The pellet was dried and resuspended in 50 μl pure water.

### Short Read Sequencing (Illumina MiSeq)

*V. cholerae* genomic DNA was isolated as described (Kemter et al., 2018) from various strains grown under different conditions (log phase vs. stationary) were quantitated using the Qubit fluorimeter (Thermo Fisher) and adjusted to 0.2 ng/μL with nuclease-free water. Sequencing libraries were prepared using the Illumina Nextera XT kit, processed and pooled according to manufacturer’s instructions (Illumina). The final, pooled sample was paired-end sequenced (2×300 bp) using the Illumina MiSeq with a v3 chemistry, 600 cycle kit. Post sequencing processing was performed the systems software packages and the final demultiplexed fastq reads produced by the instrument were used for MFA against the reference genome. Raw sequencing data are available on request.

### Long Read Sequencing (PacBio)

Whole genome sequencing was performed on a Pacific Biosciences RSII platform. The sequencing library was prepared using the SMRTbell^TM^ Template Prep Kit (Pacific Biosciences, Menlo Park, CA, United States) following manufacturer’s protocol. 5 μg of DNA was fragmented using gTUBE (Covaris Inc., Woburn, MA, United States) to ∼20 kb. After DNA damage repair and ends repair, blunt hairpin adapters were ligated to the template. Non-ligated products were digested by ExoIII and ExoVII exonucleases. Resulting SMRTbell template were purified with AMPure PB beads and size selected on BluePippin system (Sage Science, Beverly, MA, United States), using 0.75% dye-free agarose cassette, with 4–10 kb Hi-Pass protocol and lower cut set on 4 kb. Size selected purified libraries were quantified by Qubit dsDNA High Sensitivity assay. After primer annealing, and P6 polymerase binding, templates were bound to MagBeads for loading. Each sample was sequenced on two SMRT cells, using C4 sequencing kit and 360-min movies per SMRT cell. Presence of unidentified contaminant in two libraries (NSV2 16 days) inhibited sequencing reactions, which manifested in extremely low P1 (2%) and super short reads. Both libraries were subjected to the cleanup procedure that involves binding annealed SMRTbell libraries to magnetic beads, washing the bound annealed DNA SMRTbell templates to remove potential contaminants, and eluting the purified, annealed DNA SMRTbell templates from the magnetic beads. The purified SMRTbell templates were then re-quantified by Qubit and prepared for sequencing on the PacBio RSII according to the Binding Calculator. After cleanup procedure, PacBio RS II instrument sequencing yields were comparable to the other samples. Raw sequencing data are available on request.

### Marker Frequency Analysis

Sequencing reads from a NGS were mapped to the respective genome using the program Geneious (Biomatters Ltd.; Kearse et al., 2012). Read densities were extracted and plotted using custom R scripts as described previously (Kemter et al., 2018).

### Semiquantitative Conjugation

All used replicons possess an *oriR6K* and were conjugated from *E. coli strain* WM3064 to MG1655. For overnight cultures of donor and recipient strains the OD600 was determined and the amount of cells corresponding to 1 ml of OD600 = 1 was centrifuged 1 min at 13,000 ×*g*. The cells were washed twice in TBS and resuspended in 100 μl TBS. From each donor strain, 50 μl were mixed with 50 μl of the recipient strain and dropped on LB agar including DAP. After 6 h cells were scraped off the plate, washed twice in TBS. The total CFU of recipient cells was determined by plating dilutions on LB, while the CFU of plasmid bearing recipients was determined by plating the same dilutions on selective media. The selective CFU was then normalized to total CFU.

### Microscopy

Differential interference contrast (DIC) microscopy was performed on 1% (w/v) agarose pads in PBS buffer using the Nikon Ti fluorescence microscope (100× objective, NA 1.45).

### Quantification of Replicon Copy Number via Antibiotic Sensitivity

The copy-up effect of *crtS* was measured as described (Messerschmidt et al., 2016). Cells were grown in LB medium with either 100 or 500 μg/ml ampicillin at 37°C in 96-well plates in a microplate reader (Infinite M200 pro multimode microplate reader, Tecan). The main culture (150 μL) was inoculated 1:1,000 and growth curves recorded for 15 h. For better visualization, 1 divided by the time needed to reach an OD_600_ of 0.1 was defined as measure of the copy number.

## Author Contributions

MB, DS, SS, and TW contributed to the conceptualization of the study. MB, DS, FK, MW, KC, GK, and GP contributed to the methodology. MB, DS, KC, and GK contributed to the software. MB, DS, FK, MW, KC, GK, GP, SS, and TW contributed to the formal analysis of the study. MB, DS, FK, MW, KC, GK, and GP executed the investigation for the study. MB, SS, and TW contributed to the writing of the original draft. SS and TW reviewed and edited the manuscript. DS, SS, and TW provided the supervision. SS, GP, and TW contributed to the funding acquisition.

## Conflict of Interest Statement

The authors declare that the research was conducted in the absence of any commercial or financial relationships that could be construed as a potential conflict of interest.
