# Supplementary material for: Functionality of Two Origins of Replication in Vibrio cholerae Strains With a Single Chromosome
Source: Front Microbiol. 2018 Nov 30;9:2932. doi: 10.3389/fmicb.2018.02932 (PMC6284228; doi:10.3389/fmicb.2018.02932)
Supplement: Supplementary file 1 [file Data_Sheet_1.PDF]

Supplementary Material

Functionality of Two Origins of Replication in *Vibrio cholerae* Strains with a Single Chromosome

Matthias Bruhn[1], Daniel Schindler[2], Franziska S. Kemter[1], Michael R. Wiley[3], Kitty Chase[3], Galina I. Koroleva[3], Gustavo Palacios[3], Shanmuga Sozhamannan[4, 5], Torsten Waldminghaus[1]**

*Correspondence:

Torsten Waldminghaus

Torsten.Waldminghaus@SYNMIKRO.Uni-Marburg.de

Shanmuga Sozhamannan

Shanmuga.Sozhamannan.ctr@mail.mil

Supplementary tables

Supporting S1 table: Strains used in this study

Strain	Characteristics	Resistance	Reference
Escherichia coli			
MG1655	Wild type		(1)
AB330	*cf*. DY330 *lacZ+ gal+*		Alexander Böhm
KS0003	MG1655<br>dam16::Kan	KmR	(2)
WM3064	*hsdS lacZΔM15 RP4-1360 Δ(araBAD)567ΔdapA1341::[erm pir(wt)]*	EmR	William Metcalf
S17-1 *λpir*	*(F-) RP4-2-Tc::Mu aphA::Tn7recA λpir lysogen*	SmR, TpR	(3)
Top10	F- *mcr*A Δ(*mrr-hsdRMS-mcrBC*) Φ80*lac*ZΔM15 Δ*lacX74 recA1 araD139* Δ(*ara leu*) 7697 *galU galK rpsL* (StrR) *end*A1 *nupG*	StrR	Invitrogen
NZ140	MG1655 *crtS*$_{V.\ cholerae\ N16961}$ pMA568 (synVicII)	AmpR	(4)
FSK103	MG1655 *crtS*$_{V.\ cholerae\ NSCV1}$		This study
FSK104	MG1655 *crtS*$_{V.\ cholerae\ NSCV2}$		This study
FSK130	FSK103 pMA568 (synVicII)	AmpR	This study
FSK131	FSK104 pMA568 (synVicII)	AmpR	This study

Vibrio cholerae			
NSCV1	Wild type, other names: 1154-74 (Serogroup O49); VAA		(5, 6)
NSCV2	Wild type, other names: 10432-62 (Serogroup O27), VAB		(5, 6)
N16961	Wild type		(7)
MCH1	Synthetic fusion of Chr1 and Chr2 in *V. cholerae* N16961		(8)
VC49	*V. cholerae* MCH1 ChapR, natural competent	GentR	This study
VC61	*V. cholerae* VC49 with *ori2* at NSCV1 position	CmR	This study
VC62	*V. cholerae* VC49 with *ori2* at NSCV2 position	CmR	This study
VC71	*V. cholerae* VC62 *ΔcrtS*		This study
VC73	*V. cholerae* VC71 with *crtS* at NSCV2 position		This study

Supporting S2 table: Replicons used in this study

**Plasmid**	**Characteristics/ Construction**	**Resistance**	**Reference**
pICH50914	MoClo Endlinker	AmpR	(9)
pICH50927	MoClo Endlinker	AmpR	(9)
pBR-flp	FLP+, λ cI857+, λ ρR, AmpR,TcR	AmpR, TetR	(10)
pUXBF13	Transposase	AmpR	(11)
pGP704-mTn7-hapR_ATN	*hapR* on Transposon	AmpR	(12)
pMA135	*oriR6K* only	AmpR	(13)
pMA329	MoClo Lvl P vector	SpecR	(14)
pMA350	MoClo Lvl 1 vector	AmpR	(15)
pMA351	MoClo Lvl 1 vector	AmpR	(15)
pMA352	MoClo Lvl 1 vector	AmpR	(15)
pMA353	MoClo Lvl 1 vector	AmpR	(15)
pMA449	MoClo backbone: pMA349<br>Insert: PCR with primers 1439/1440 from gDNA of NSCV1<br>$crtS_{V.\ cholerae\ NSCV1}$	AmpR	This work
pMA450	MoClo backbone: pMA349<br>Insert: PCR with primers 1439/1440 from gDNA of NSCV2<br>$crtS_{V.\ cholerae\ NSCV2}$	AmpR	This work
pMA452	MoClo backbone: pMA327:<br>Insert: pMA709 + pMA710 + pMA431 + pMA449 + pICH50900	KmR, SpecR	This work
pMA453	MoClo backbone: pMA327:<br>Insert: pMA709 + pMA710 + pMA431 + pMA450 + pICH50900	KmR, SpecR	This work
pMA568	*ori2* from *V. cholerae* N16961	AmpR	(4)
pMA730	MoClo backbone: pMA350<br>Insert: PCR with primers 1206/1207 + 1208/1209 on N16961 gDNA<br>5’ flank NSCV1 position	AmpR	This work

pMA731	MoClo backbone: pMA353<br>Insert: PCR with primers 1210/1211 + 1212/1213 on N16961 gDNA<br>3' flank NSCV1 position	AmpR	This work
pMA732	MoClo backbone: pMA350<br>Insert: PCR with primers 1214/1215 on N16961 gDNA<br>5' flank NSCV2 position	AmpR	This work
pMA733	MoClo backbone: pMA352<br>Insert: PCR with primers 1216/1217 on N16961 gDNA<br>3' flank NSCV2 position	AmpR	This work
pMA734	MoClo backbone: pMA352<br>Insert: PCR with primers 703/704 on pMA404<br>FRT-Cat-FRT	AmpR, CmR	This work
pMA735	MoClo backbone: pMA329<br>Insert: pMA730+ pMA740+ pMA734+ pMA731+pICH50927<br>*ori2* NSCV1 position insertion construct	SpecR, CmR	This work
pMA736	MoClo backbone: pMA329<br>Insert: pMA732+ pMA740+ pMA734+ pMA733+pICH50927<br>*ori2* NSCV2 position insertion construct	SpecR, CmR	This work
pMA739	*ori2* from *V. cholerae* NSCV1	AmpR	This work
pMA740	MoClo backbone: pMA351<br>Insert: PCR with primers 1204/1205 on pMA650<br>*ori2* from *V. cholerae*	AmpR	This work
pMA741	MoClo backbone: pMA350<br>Insert: PCR with primers 1445/1446 on N16961 gDNA<br>5' flank *crtS* insertion	AmpR	This work
pMA742	MoClo backbone: pMA350<br>Insert: PCR with primers 1449/1450 on N16961 gDNA<br>5' flank *crtS* deletion	AmpR	This work
pMA743	MoClo backbone: pMA351<br>Insert: PCR with primers 1457/1458 on N16961 gDNA<br>*crtS*	AmpR	This work
pMA744	MoClo backbone: pMA351<br>Insert: PCR with primers 1455/1456 on pKD4<br>FRT-Kan-FRT	AmpR, KanR	This work
pMA745	MoClo backbone: pMA352<br>Insert: PCR with primers 1455/1456 on pKD4<br>FRT-Kan-FRT	AmpR, KanR	This work
pMA746	MoClo backbone: pMA352<br>Insert: PCR with primers 1451/1452 + 1453/1454 on N16961 gDNA<br>3' flank *crtS* deletion	AmpR	This work

pMA747	MoClo backbone: pMA353<br>Insert: PCR with primers 1447/1448 on N16961 gDNA<br>3’ flank *crtS* insertion	AmpR	This work
pMA748	MoClo backbone: pMA329<br>Insert: pMA742 + pMA744 + pMA746 + pICH50914<br>*crtS* deletion construct	SpecR, KanR	This work
pMA749	MoClo backbone: pMA329<br>Insert: pMA741 + pMA743 + pMA745 + pMA747 + pICH50927<br>*crtS* NSCV2 position insertion construct	SpecR, KanR	This work
pMA755	*ori2* from *V. cholerae* NSCV2	AmpR	This work
pMA899	*oriF*	AmpR	(4)

Supporting S3 table: Oligonucleotides used in this study

Name	Sequence (5´ » 3´)
703	AAGGTCTCGGGAGGAAGTTCCTATACTTTCTAGAGAATAGGAACTTCGGAATAGGAACTTCATTTAAATGG
704	TTGGTCTCCAGCGAAGTTCCTATTCTCTAGAAAGTATAGGAACTTCGGCGCGCCTACCTGTGACGG
1204	AAGGTCTCGGGAGCTAAAACGCACAAAGCCCG
1205	TTGGTCTCCAGCGGTAGGCAAAAAAAGAGCGAGC
1206	AAGGTCTCGGGAGTTGCTAGGTGTGATGTCGAAAAATATCG
1207	TTGGTCTCCAGGATCTTCTGGCTTACCAC
1208	AAGGTCTCGTCCTATGCCACAAGCGG
1209	TTGGTCTCCAGCGGAGTGAGAGTTTTGTCCACG
1210	AAGGTCTCGGGAGTTTAATAAAGAAGCGACCATTGGTCGCC
1211	TTGGTCTCCAACTCAGCAGTACCACACGC
1212	AAGGTCTCGAGTTTTCAGCAAGCCTGCAA
1213	TTGGTCTCCAGCGCAGGATTCTCTCCAACGCC
1214	AAGGTCTCGGGAGGGTAGATAAATGAGCGAAAAGTTACAAAAAG
1215	TTGGTCTCCAGCGACATCGCTCGAAGAAAGGC
1216	AAGGTCTCGGGAGCGCTTATAAAAAAAGCCAGTCATGTTG
1217	TTGGTCTCCAGCGGCCAGCGTCGAGTTATTGATG
1227	GAACTTCCTCGAGCATCTCCGACGGGTCGGGATTTTGCCAAATCGTAG
1228	AATAAACAAATAGGGGTTCCGCGGGCTAAAACGCACAAAGCCCGC
1439	AAGGTCTCGGGAGTGAGTGATCTTTTCACTATATTC
1440	TTGGTCTCCAGCGCTCTTTTAAGCAGGATCCTAAC
1449	AAGGTCTCGGGAGCCCATTTGAAGGTAAGACG
1450	TTGGTCTCCAGCGGATTCTGAGGGCTGCGC
1451	AAGGTCTCGGGAGTATCAGTTATTGGTTAATTAAGTCGCATCG
1452	AAGGTCTCGGAAAGACAAAACGGATTTTGGATTTCG
1453	AAGGTCTCGTTTCAACCGCTTAACGGGAATTCAC
1454	TTGGTCTCCAGCGGTACTATCGCTGCGAGTGAAATC
1455	AAGGTCTCGGGAGGAAGTTCCTATACTTTCTAGAGAATAGGAACTTCGGAATAGGAACTTCAAGATCCCC
1456	TTGGTCTCCAGCGAGTTCCTATTCCGAAGTTCCTATTCTCTAGAAAGTATAGGAACTTCAGAGCGCTTTTG

1445	AAGGTCTCGGGAGGTGCTTTTCCGTTTGTGCAG
1446	TTGGTCTCCAGCGGTCAAACAGGCAGCGC
1447	AAGGTCTCGGGAGGAACTGAGCATTTATATACAGCC
1448	TTGGTCTCCAGCGCAACGTGGAAACGGTTG
1457	AAGGTCTCGGGAGTGAGTGATCTTTTCACTATATTCTGTCTTAAG
1458	TTGGTCTCCAGCGCTCTTTTAAGCAGGATCCTAACCG

Supporting S4 table: Doubling times of natural and engineered *V. cholerae* strains

Strain	Doubling time (min)	Standard deviation (min)
N16961	16	0.2
NSCV1	20	0.5
NSCV2	29	1.3
VC61	26	0.1
VC62	27	0.5
VC71	25	0.6
VC73	26	0.2

Supplementary figure

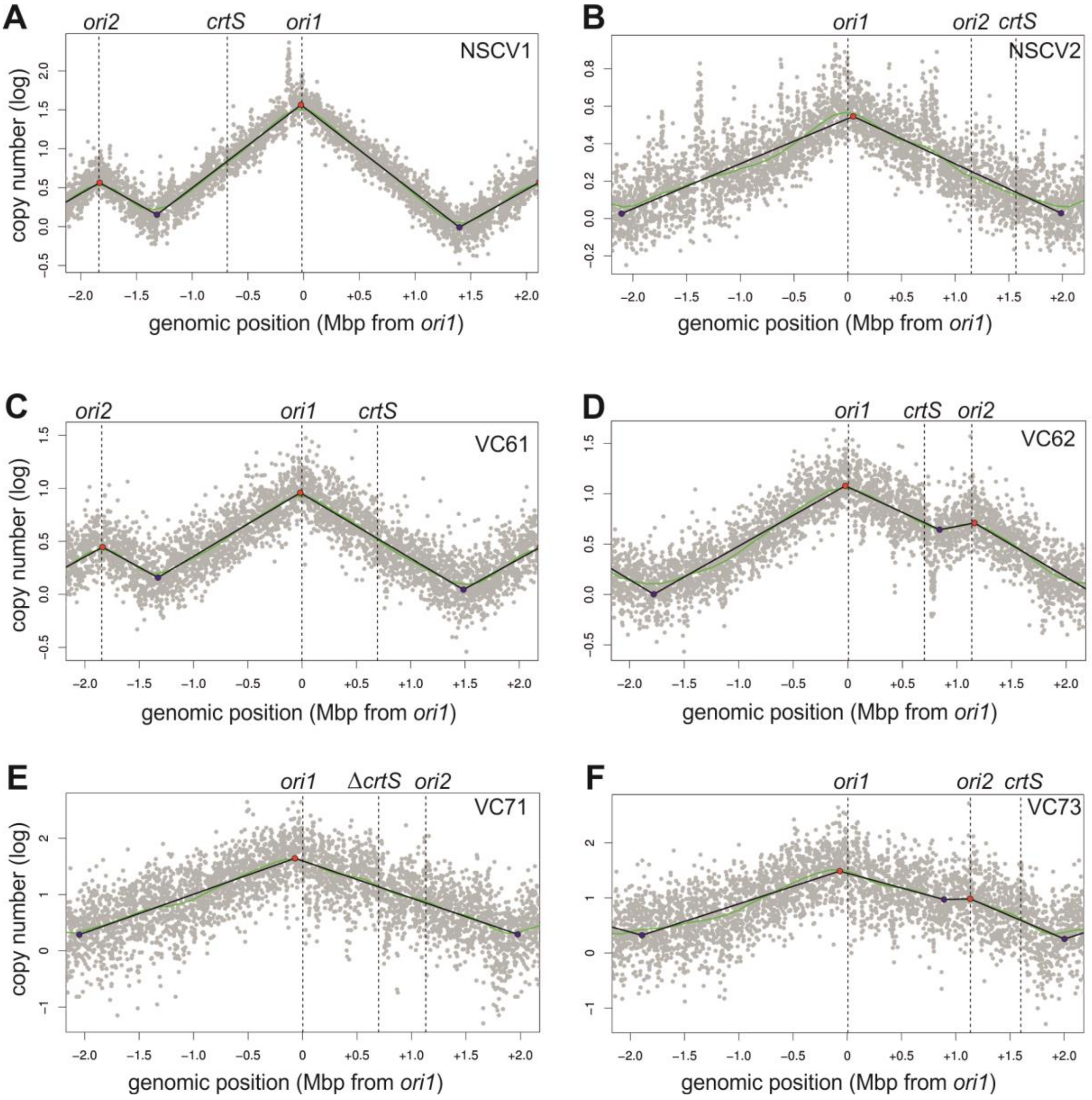

Supplementary figure S1. Biological replicates of Marker Frequency Analysis (MFA) of *V. cholerae* strains. Replicates are shown for indicated strains as replicates of data shown in fig. 3, 5, 7 and 8. Grey dots represent log numbers of normalized reads as mean values for 1 kbp windows relative to the stationary phase sample. The genome position is shown as the distance from *ori1*. Vertical dotted black lines mark the locations of replication origins and the *crtS* site. The solid black lines represent the fitting of regression lines and the green line corresponds to the Loess regression (F = 0.05). Maxima are highlighted by red and minima as blue dots.

Supplementary references

1. Blattner FR, Plunkett G, 3rd, Bloch CA, Perna NT, Burland V, Riley M, Collado-Vides J, Glasner JD, Rode CK, Mayhew GF, Gregor J, Davis NW, Kirkpatrick HA, Goeden MA, Rose DJ, Mau B, Shao Y. 1997. The complete genome sequence of *Escherichia coli* K-12. Science 277:1453-62.
2. Skarstad K, Lobner-Olesen A. 2003. Stable co-existence of separate replicons in *Escherichia coli* is dependent on once-per-cell-cycle initiation. EMBO J 22:140-50.
3. Simon R, Priefer U, Puhler A. 1983. A Broad Host Range Mobilization System for Invivo Genetic-Engineering - Transposon Mutagenesis in Gram-Negative Bacteria. Bio-Technology 1:784-791.
4. Schallopp N, Milbredt S, Sperlea T, Kemter FS, Bruhn M, Schindler D, Waldminghaus T. 2017. Establishing a System for Testing Replication Inhibition of the Vibrio cholerae Secondary Chromosome in Escherichia coli. Antibiotics (Basel) 7.
5. Johnson SL, Khiani A, Bishop-Lilly KA, Chapman C, Patel M, Verratti K, Teshima H, Munk AC, Bruce DC, Han CS, Xie G, Davenport KW, Chain P, Sozhamannan S. 2015. Complete Genome Assemblies for Two Single-Chromosome *Vibrio cholerae* Isolates, Strains 1154-74 (Serogroup O49) and 10432-62 (Serogroup O27). Genome Announc 3.
6. Xie G, Johnson SL, Davenport KW, Rajavel M, Waldminghaus T, Detter JC, Chain PS, Sozhamannan S. 2017. Exception to the Rule: Genomic Characterization of Naturally Occurring Unusual Vibrio cholerae Strains with a Single Chromosome. Int J Genomics 2017:8724304.
7. Heidelberg JF, Eisen JA, Nelson WC, Clayton RA, Gwinn ML, Dodson RJ, Haft DH, Hickey EK, Peterson JD, Umayam L, Gill SR, Nelson KE, Read TD, Tettelin H, Richardson D, Ermolaeva MD, Vamathevan J, Bass S, Qin H, Dragoi I, Sellers P, McDonald L, Utterback T, Fleishmann RD, Nierman WC, White O, Salzberg SL, Smith HO, Colwell RR, Mekalanos JJ, Venter JC, Fraser CM. 2000. DNA sequence of both chromosomes of the cholera pathogen *Vibrio cholerae*. Nature 406:477-83.
8. Val ME, Skovgaard O, Ducos-Galand M, Bland MJ, Mazel D. 2012. Genome engineering in Vibrio cholerae: a feasible approach to address biological issues. PLoS Genet 8:e1002472.
9. Weber E, Engler C, Gruetzner R, Werner S, Marillonnet S. 2011. A modular cloning system for standardized assembly of multigene constructs. PLoS One 6:e16765.
10. De Souza Silva O, Blokesch M. 2010. Genetic manipulation of *Vibrio cholerae* by combining natural transformation with FLP recombination. Plasmid 64:186-95.
11. Bao Y, Lies DP, Fu H, Roberts GP. 1991. An improved Tn7-based system for the single-copy insertion of cloned genes into chromosomes of gram-negative bacteria. Gene 109:167-8.
12. Meibom KL, Blokesch M, Dolganov NA, Wu CY, Schoolnik GK. 2005. Chitin induces natural competence in *Vibrio cholerae*. Science 310:1824-7.
13. Messerschmidt SJ, Schindler D, Zumkeller CM, Kemter FS, Schallopp N, Waldminghaus T. 2016. Optimization and Characterization of the Synthetic Secondary Chromosome *synVicII* in *Escherichia coli*. Front Bioeng Biotechnol 4:96.
14. Schindler D, Milbredt S, Sperlea T, Waldminghaus T. 2016. Design and Assembly of DNA Sequence Libraries for Chromosomal Insertion in Bacteria Based on a Set of Modified MoClo Vectors. ACS Synth Biol 5:1362-1368.
15. Zumkeller CM, Schindler D, Waldminghaus T. 2018. Modular assembly of synthetic secondary chromosomes. Methods in Molecular Biology.
